# A novel chromosome 2q24.3‐q32.1 microdeletion in a fetus with multiple malformations

**DOI:** 10.1002/jcla.24602

**Published:** 2022-07-12

**Authors:** Mianmian Zhu, Yihong Wang, Lijie Guan, Chaosheng Lu, Rongyue Sun, Yuan Chen, Jiamin Shi, Yanying Zhu, Dan Wang

**Affiliations:** ^1^ Department of Pediatrics The First Affiliated Hospital of Wenzhou Medical University Wenzhou China; ^2^ Department of Ultrasound imaging The First Affiliated Hospital of Wenzhou Medical University Wenzhou China; ^3^ Clinical Laboratory The First Affiliated Hospital of Wenzhou Medical University Wenzhou China

**Keywords:** 2q deletion, de novo, *HOXD13*, microdeletion, multiple congenital anomalies

## Abstract

**Background:**

Terminal or interstitial deletion of chromosome 2q is rarely reported but clinically significant, which can result in developmental malformations and psychomotor retardation in humans. In the present study, we analyzed this deletion to comprehensively clarify the relationship between phenotype and microdeletion region.

**Methods:**

We collected clinical records of the fetus and summarized patient symptoms. Subsequently, genomic DNA was extracted from fetal tissue or peripheral blood collected from parents. In addition, whole‐exome sequencing (WES) and copy number variation sequencing (CNV‐seq) were performed.

**Results:**

The fetus presented a previously unreported interstitial deletion of 2q24.3‐q32.1. WES and CNV‐seq revealed a de novo 18.46 Mb deletion at 2q24.3‐q32.1, a region involving 94 protein‐coding genes, including *HOXD13, MAP3K20, DLX1, DLX2, SCN2A*, and *SCN1A*. The fetus had upper and lower limb malformations, including camptodactyly and syndactyly, along with congenital cardiac defects.

**Conclusion:**

Herein, we report a fetus with a novel microdeletion of chromosome 2q24.3‐q32.1, likely a heterozygous pathogenic variant. Haploinsufficiency of *HOXD13* might be related to limb deformity in the fetus.

## Graphical abstract

Terminal or interstitial deletion of chromosome 2q is rarely reported but clinically significant. There was an interstitial deletion of 2q24.3‐q32.1 in the fetus. WES and CNV‐seq revealed a *de novo* 18.46Mb deletion at 2q24.3‐q32.1, a region involving 94 protein‐coding genes, including *HOXD13, MAP3K20, DLX1, DLX2, SCN2A* and *SCN1A*.

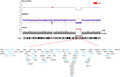

## 1 INTRODUCTION

The long arm of chromosome 2 is unique in human autosomes, originating from the head‐to‐head fusion of two ancestral chromosomes at 2q13 with the ancestral centromere at 2q21.^1^ Terminal or interstitial deletion of the long arm of chromosome 2 is a rare copy number variations (CNVs), with approximately 100 cases reported in available literature.^2^ Furthermore, this deletion has been associated with epilepsy, intellectual disability, developmental delay, cardiovascular malformation, hypospadias and cryptorchidism, digital abnormalities, and other visceral organ anomalies.^3^ Clinical manifestations vary greatly based on the size and location of the deletion.

A deletion involving 2q24.3 has been previously reported, and the patient exhibited psychomotor retardation, low set ears, cranial sutural irregularities, and laryngomalacia.^4^ Microdeletion of 2q31.1 is deemed a clinically recognizable gene syndrome characterized by short stature, moderate‐to‐severe developmental delay, microcephaly, hypotonia, specific craniofacial dysmorphisms, and upper/lower limb deformities associated with *HOXD* genes.^5^ Previously, chromosome deletions were discovered by Giemsa banding.^6^ Chromosome deletions spanning over 5 Mb are microscopically visualized on chromosome‐banded karyotypes. Given the development of next‐generation sequencing technology, CNV sequencing (CNV‐seq) has been widely employed in recent years. Compared with conventional methodology, CNV‐seq has advantages such as high throughput, high resolution, and relatively low cost.^7^ Moreover, CNV‐seq can detect deletions above 100 Kb.

Herein, we describe a novel interstitial heterozygous deletion that encompasses the 2q24.3‐q32.1 chromosomal region, as determined using CNV‐seq and whole exosome sequencing (WES). The deletion was found to affect 94 genes, of which 33 are associated with diseases, including *HOXD13*, *MAP3K20*, *DLX1*, *DLX2*, *SCN2A*, and *SCN1A*. We analyzed the clinical features and genes on the deletion region to further interpret the relationship between the deletion region and phenotype.^8^

## 2 METHODS

### 2.1 Participants

The proband and parents were enrolled at The First Affiliated Hospital of Wenzhou Medical University. Written consent was obtained from the parents of the fetus prior to commencing the study. All study protocols were reviewed and approved by the ethics committees of The First Affiliated Hospital of Wenzhou Medical University. Relevant clinical records (symptoms, appearance and duration of symptoms, physical and ultrasound examination) were collected and examined.

### 2.2 DNA extraction

According to the manufacturer's standard instructions, genomic DNA was extracted from the fetal muscle and his parents' peripheral blood samples conserved in EDTA using the Tissue Genome DNA Extraction Kit DP341 and Blood Genome DNA Extraction Kit DP329 (TianGen). DNA purity and concentration were determined using the Nanodrop ND‐1000 Spectrophotometer (Thermo Fisher Scientific). Genomic DNA was stored at −20°C until use.

### 2.3 WES

Briefly, ultrasound was used to break genomic DNA into 250–300 bp fragments. DNA libraries were constructed by end filling, adapter ligation, and polymerase chain reaction amplification.^9^ Then, the DNA libraries underwent hybridization capture and were enriched by the xGen Exome Research Panel v2.0 (IDT). High throughput sequencing was performed on the DNBSEQ‐T7 platform (Beijing Genomics Institute). After filtration and quality control, clean reads were aligned to the University of California Santa Cruz (UCSC) human reference genome (hg19) using the Burrows‐Wheeler mapping algorithm.^10^ Combined with OMIM, HGMD, SwissVar, Clinvar, and dbSNP, the genetic variation was analyzed, classified, and annotated with the American College of Medical Genetics (ACMG).

### 2.4 CNV‐seq

The DNA libraries were single‐ended sequenced on the DNBSEQ‐T7 platform (Beijing Genomics Institute), with a sequencing depth of 0.2x. Raw sequencing reads were processed according to the quality control standards and subsequently compared with the hg19 of the UCSC using Burrows‐Wheeler Alignment.^10^ Using read counts, Z‐scores, and log2Ratio, the in‐house bioinformatics pipeline evaluated CNVs.^7^ The candidate CNVs were filtered with the Accurate Diagnosis of Genetic Diseases Cloud Platform (Quanpu). Subsequently, CNVs were annotated based on the publicly available databases, including Decipher, Clinvar, ISCA, OMIM, ClinGen and UCSC (http://genome.ucsc.edu).^11^ Finally, according to the ACMG guidelines, CNVs were divided into five categories: pathogenic, likely pathogenic, likely benign, uncertain clinical significance, and benign.^12^

## 3 RESULTS

### 3.1 Clinical data

**FIGURE 1:**
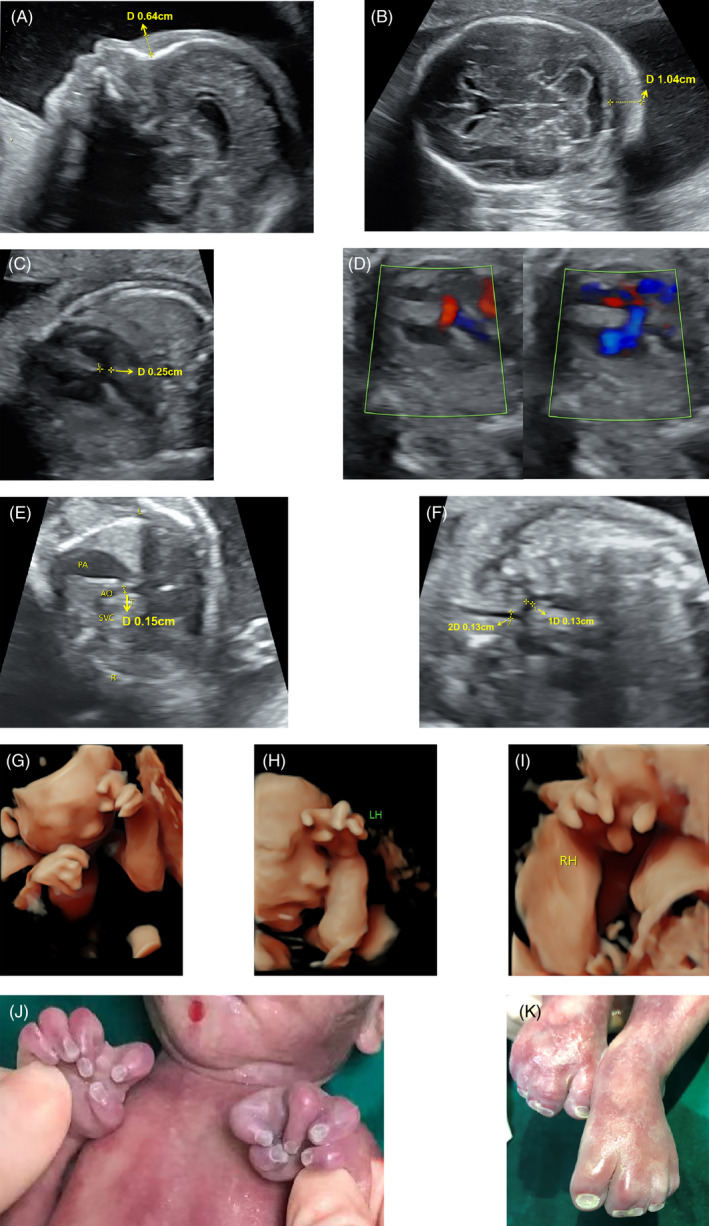
The ultrasound image of the fetus. (A) The anterior nasal skin is approximately 0.64 cm thick. (B) The nuchal fold is thickened to 1.04 cm. (C) The continuity of the ventricular septum is interrupted by approximately 0.25 cm. (D) Color Doppler flow imaging shows a bidirectional shunt on the ventricular level. (E) In the three‐vessel and trachea view, the ascending aorta is significantly narrower than the pulmonary artery. The transverse aortic arch is 0.15 cm wide. (F) The inner diameter of the aortic isthmus is 0.13 cm. (G–J) A wide gap between the thumb, index finger, and middle finger. Camptodactyly. Proximally placed fourth finger. (K) Syndactyly between second and third toes

The male fetus (the proband) was the second child of young and non‐consanguineous parents. The maternal pregnancy was uncomplicated. Family history included a spontaneous abortion (embryo arrest) at 8 weeks of gestation. No consanguinity was reported. Prenatal care showed no history of exposure to radiation and toxic agents. At the 23rd week of gestation, the fetus exhibited increased anterior nasal skin and nuchal fold (Figure 1A and B). The echocardiogram indicated ventricular septal defect and aortic dysplasia (Figure 1C–F). A routine prenatal ultrasound revealed abnormal fetal hand posture and an increased distance between fingers (Figure 1G–I). In addition, the gallbladder was unclear on the ultrasound image, along with the presence of polyhydramnios. The pregnancy was terminated, and the fetus was aborted owing to multiple malformations at 27 weeks of gestation, with a weight of 875 g (10th–25th percentile), length of 35 cm (25th–50th percentile), and a head circumference of 23.5 cm (10th–25th percentile). On physical examination, the fetus exhibited dysmorphic features, including proximally placed fourth finger and camptodactyly. As shown in the ultrasonic image, the distance between the thumb, index finger, and middle finger was increased, with splaying between the index and middle fingers (Figure 1J). His feet were symmetrical with complete cutaneous syndactyly of the second and third digits (Figure 1K). The necropsy was refused.

### 3.2 CNVs detection

**FIGURE 2:**
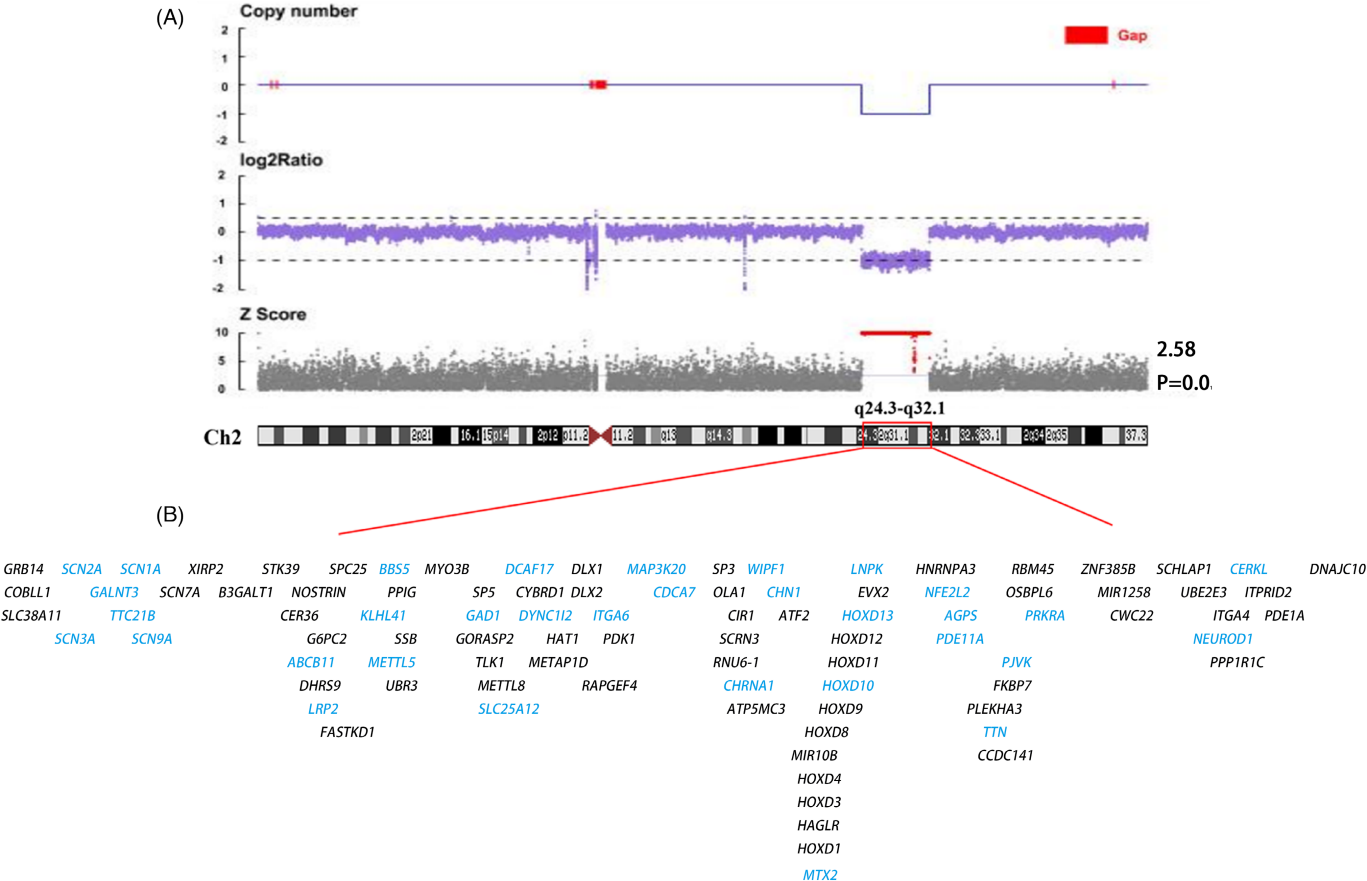
Copy number variations (CNVs) detection. (A) CNV sequencing shows an 18.46 Mb deletion circled in red. The interstitial deletion is at chromosome 2q24.3–32.1. (B) The protein‐coding genes are located in the deleted region. The genes marked in blue are related to the disease

WES revealed a heterozygous deletion at genomic position (chr2: 165125352–183,581,904) (Assembly hg19). CNV‐seq confirmed the likely 18.46 Mb pathogenic CNVs on chromosome 2 (Figure 2A). This position corresponded to the 2q24.3 and 2q32.1 cytogenetic bands. The chromosomal constitution was as follows: 46,XY array2q24.3q32.1 (165125352–183581904) × 1. The deletion affected 94 protein‐coding genes, including *HOXD13*, *MAP3K20*, *DLX1*, *DLX2*, *SCN2A*, and *SCN1A* (Figure 2B). Both parents did not carry abnormal CNVs, which indicated that the deletion in the proband was de novo.

## 4 DISCUSSION

Herein, the proband presented camptodactyly, syndactyly, proximally placed fourth finger, ventricular septal defect, and aortic dysplasia. We identified a novel heterozygous interstitial deletion at chromosome 2q24.3–32.1 (chr2: 165125352–183581904), which could have markedly contributed to the fetal phenotype. The deletion involved 94 protein‐coding genes, including 33 morbid genes related to recognizable clinical phenotypes. Among these, *HOXD13*, *SCN2A*, and *SCN1A* have exhibited haploinsufficiency in ClinGen.^13^ Table 1 summarizes the clinical features of patients with chromosome deletion from 2q24.3 to 32.1.^3^, ^6^, ^14^, ^15^, ^16^, ^17^

**TABLE 1:** Clinical features of chromosome deletion from 2q24.3 to 32.1

Phenotype	Lazier et al.	Tsai et al.	Svensson et al.	Pescucci et al.	Boles et al.	Dimitrov et al. [*n* = 5]	Our case
Start‐end	2q24.3‐q31.1	2q31.1‐31.2	2q31.1	2q24.3‐q31.1	2q24.2‐q31.1	2q24.3‐q32.1^b^	2q24.3‐q32.1
Size (Mb)	10.4	3.4	2.518	10.4	NS	2.74–16.9	18.46
Gender	Female	Female	Female	Female	Male	1 M: 4 F	Male
Birth Height	NS	NS	10th–25th	25th–50th	NS	2/5[NBW] (1/5 NS)	25th–50th
Birth Weight	10th	<3th	50th–75th	10th–25th	2890 g	3/5[NBW] (1/5 NS)	10th–25th
Postnatal developmental retardation	+	+	+	+	+	+	NA
microcephaly	+	+^a^	+	+	+	2/5	−
Cranial sutural irregularities	+	−	−	−	−	2/5	−
ptosis/epicanthus	+	−	−	+	+	4/5	−
Low set/dysplastic ears	−	+	+	+	+	2/5	−
Bilateral limb deformity	+	+	+	NS	+	4/5(1/5 NS)	+
Syndactyly	+	−	+	+	+	3/5	+
Camptodactyly	+	−	−	−	+	1/5	+
Wide gap between digits	+	−	−	+	+	2/5	+
Clinodactyly	+^a^	+	+	+	−	3/5	+
Tapering fingers	−	+	−	+	−	1/5	−
Wide halluces	−	+	−	+	+	1/5	−
Cardiac anomalies	−	−	−	−	+	2/5	+
Strabismus	+	−	+	−	−	1/5	−

*Note*: NBW, normal birth weight; NS, not specified; NA, not applicable; +, present; −, absent.

^a^ Extrapolated based on descriptive features.

^b^ Start‐end: patient1, 2q24.3‐q31; patient2, 2q31.1‐q32.1; patient3, 2q31.1‐q31.2; patient4, 2q24.3‐q31.1; patient5, 2q31.1.

In this study, the most prominent feature was deformity of the upper and lower limbs, including camptodactyly, syndactyly, and clinodactyly. All reported patients with 2q24.3–32.1 deletions appear to present limb abnormalities. Overall, 8/11 patients presented syndactyly, 4/11 patients exhibited camptodactyly, and 8/11 patients presented clinodactyly. As noted in mouse mutants, the *HOXD* cluster and surrounding regulatory sequences are considered the underlying cause of the limb phenotype in this region.^3^, ^18^ Deletion, translocation, or disruption of this locus can reportedly cause camptodactyly, syndactyly, brachydactyly, ectrodactyly, and polydactyly.^14^, ^19^ Considering the current case study, the deletion contained *HOXD13*, an essential gene for regulating and developing the genital tract and autopod that forms hands and feet.^20^ In addition, it has been suggested that the *HOXD* cluster can regulate the size and number of digits in a dose‐dependent manner, indicating a negative relationship between the *HOXD* gene and digit number rather than qualitative functions.^21^, ^22^ In the presence of multiple homozygous *HOXD* mutations, major limb defects are likely to occur.^22^ Heterozygous deletion of *HOXD13* may lead to *HOXD13* haploinsufficiency. The heterozygous loss‐of‐function variants reduce the production of functional protein binding to DNA, while sustaining the basic function of *HOXD13* protein.^23^ Therefore, it exhibits a milder phenotype, similar to that observed in our proband and shows incomplete penetrance with some frequency.^24^, ^25^ Meanwhile, it explains the limb phenotypes with different reported severity.

Spielmann et al.^26^ have found that *Map3k20*, a gene within the deletion region, is expressed in developing limbs. Furthermore, the authors summarized the clinical manifestations of *Map3k20* mutations, including split‐foot malformation with mesoaxial polydactyly, which is related to hearing loss and exhibits a possible clinical phenotype of cutaneous syndactyly.^26^, ^27^ Herein, cutaneous syndactyly was an important malformation in the examined fetus. However, according to mouse experiments and reported pedigrees, the heterozygous deletion of *Map3k20* did not induce abnormal morphological changes.

Severe limb deformities, including split hand and monodactyly, have also been reported, and *DLX1* and *DLX2* are speculated to be novel candidate genes.^5^, ^14^, ^28^ However, upper and lower limb malformations in the examined fetus did not confirm this possibility. Theisen et al.^29^ have reported individuals exhibiting deleted *DLX1/DLX2* integrally, and no obvious limb phenotype was detected. In mutant mouse experiments, heterozygous/homozygous *DLX1/DLX2* knockouts did not induce limb abnormalities, but could produce marked craniofacial and spinal abnormalities.^30^ Facial dysmorphism is a well‐known feature in 2q31.1 microdeletion; however, no gene cluster has been defined. Interestingly, the examined fetus had no facial deformities, which could be attributed to the distinct expression of this gene in different species. Further experiments are warranted to determine whether *DLX1/DLX2* deletion could explain craniofacial abnormalities.

Chromosomal deletion is frequently associated with congenital heart defects (CHD) of unknown pathogenesis. Examining the echocardiogram, our proband exhibited a ventricular septal defect and aortic dysplasia. The ascending aorta was significantly narrower than the pulmonary artery in the three‐vessel and trachea view. Based on the echocardiogram, the examined fetus did not exhibit large ventricular septal defects and abnormal left ventricular development. Aortic dysplasia is primarily associated with chromosomal anomalies. Alison et al.^31^ have found that approximately 40% of patients with split hand and monodactyly mapped to chromosome 2 exhibited CHD, and *DLX* genes might affect the migration of neural crest cells to influence the formation of cardiovascular derivatives.^32^ Ventricular septal defect is the most frequently detected CHD. Overall, 4/11 patients were found to exhibit a ventricular septal defect. *TTN* is located in the deleted region, encodes titin protein, and is overexpressed in the fetal heart and skeletal muscle. The large spectrum of observed cardiologic phenotypes suggests that titin‐mediated defects (caused by *TTN* mutations) could underlie certain cardiac conditions with or without skeletal muscle involvement, such as ventricular septal defect.^33^ *TTN* mutations are also associated with dilated cardiomyopathy.^34^ In addition, *ATF‐2*, one of the deleted genes, is critical for cardiomyocyte differentiation.^35^ *ATF‐2* has been shown to regulate the expression of five genes associated with left ventricular outflow tract obstruction.^36^ Moreover, it suggests that the heterozygous *ATF‐2* deletion could lead to heart defects.

*SCN2A* and SCN1A, two genes detected in the current fetus, are known to be associated with epilepsy.^37^ Haploinsufficiency of *SCN2A* and *SCN1A* is reportedly responsible for nervous system dysfunction. *SCN1A* has been associated with several epilepsy syndromes with distinct clinical severities, especially the Dravet syndrome (DS), a refractory childhood epilepsy characterized by intractable seizures, developmental disorders, and increased mortality.^38^ The heterozygous deletion of *SCN2A* mainly induces autism spectrum disorders and intellectual disability.^39^ However, given the death of our proband, several potential symptoms could not develop, and no neurological examinations, such as cerebral magnetic resonance and electroencephalogram, could be performed. Deletion of *SCN2A* and *SCN1A* genes did induce notable clinical effects in our proband.

In summary, we report a de novo interstitial deletion of 2q24.3‐q32.1. This genomic segment involves 94 protein‐coding genes, and 33 of these are related to recognizable clinical phenotypes. This case study further supports the role of *HOXD13* haploinsufficiency in limb defects. Furthermore, we identified possible causative genes by analyzing gene function and phenotype. Certain defects may be due to the cumulative effect of genes in deleted fragments.

## CONFLICT OF INTEREST

The authors have no conflicts of interest to declare.

## Supporting information


Appendix S1

## Data Availability

The data that support the findings of this study are available from the corresponding author upon reasonable request.

## References

[1] Baldini A , Ried T , Shridhar V , et al. An alphoid DNA sequence conserved in all human and great ape chromosomes: evidence for ancient centromeric sequences at human chromosomal regions 2q21 and 9q13. Hum Genet. 1993;90(6):577‐583. 8444464 10.1007/BF00202474

[2] Almuzzaini B , Alatwi NS , Alsaif S , al Balwi MA . A novel interstitial deletion of chromosome 2q21.1‐q23.3: case report and literature review. Mol Genet Genom Med. 2020;8(4):1‐5. 10.1002/mgg3.1135 PMC7196451 31989799

[3] Dimitrov B , Balikova I , de Ravel T , et al. 2q31.1 microdeletion syndrome: redefining the associated clinical phenotype. J Med Genet. 2011;48(2):98‐104. 21068127 10.1136/jmg.2010.079491

[4] Bernar J , Sparkes RS , Allensworth S . Interstitial deletion 2q24.3: case report with high resolution banding. J Med Genet. 1985;22(3):226‐228. 4009646 10.1136/jmg.22.3.226 PMC1049430

[5] Puvabanditsin S , February M , Shaik T , Kashyap A , Bruno C , Mehta R . 2q31.1 microdeletion syndrome: case report and literature review. Clin Case Rep. 2015;3(6):357‐360. 26185628 10.1002/ccr3.260 PMC4498842

[6] Boles RG , Pober BR , Gibson LH , et al. Deletion of chromosome 2q24‐q31 causes characteristic digital anomalies: case report and review. Am J Med Genet. 1995;55(2):155‐160. 7717414 10.1002/ajmg.1320550204

[7] Qi Q , Jiang Y , Zhou X , et al. Simultaneous detection of CNVs and SNVs improves the diagnostic yield of fetuses with ultrasound anomalies and normal karyotypes. Genes (Basel). 2020;11(12):1397. 10.3390/genes11121397 PMC7759943 33255631

[8] Tung Y , Lu H , Lin W , et al. Case report: identification of a de novo microdeletion 1q44 in a patient with seizures and developmental delay. Front Genet. 2021;12:648351. 34093647 10.3389/fgene.2021.648351 PMC8173053

[9] Zhang J , Zhang B , Liu T , Xie H , Zhai J . Partial trisomy 4q and monosomy 5p inherited from a maternal translocationt(4;5)(q33; p15) in three adverse pregnancies. Mol Cytogenet. 2020;13(1):26. 32625247 10.1186/s13039-020-00492-4 PMC7329393

[10] Liu J , Wang K , Li B , Yang X . A novel Xp11.22–22.33 deletion suggesting a possible mechanism of congenital cervical spinal muscular atrophy. Mol Genet Genom Med. 2021;9(3):e1606. 10.1002/mgg3.1606 PMC8104167 33513289

[11] Kent Wj SCFT . The human genome browser at UCSC. Genome Res. 2002;12(6):996‐1006. 12045153 10.1101/gr.229102 PMC186604

[12] Kearney HM , Thorland EC , Brown KK , Quintero‐Rivera F , South ST . American College of Medical Genetics standards and guidelines for interpretation and reporting of postnatal constitutional copy number variants. Genet Med. 2011;13(7):680‐685. 21681106 10.1097/GIM.0b013e3182217a3a

[13] Thaxton C , Good ME , Distefano MT , et al. Utilizing ClinGen gene‐disease validity and dosage sensitivity curations to inform variant classification. Hum Mutat. 2021. doi:10.1002/humu.24291. Online ahead of print. PMC9035475 34694049

[14] Lazier J , Chernos J , Lowry RB . A 2q24.3q31.1 microdeletion found in a patient with Filippi‐like syndrome phenotype: a case report. Am J Med Genet A. 2014;164(9):2385‐2387. 10.1002/ajmg.a.36636 24924433

[15] Tsai L , Liao H , Chen Y , et al. A novel microdeletion at chromosome 2q31.1‐31.2 in a three‐generation family presenting duplication of great toes with clinodactyly. Clin Genet. 2009;75(5):449‐456. 19459884 10.1111/j.1399-0004.2008.01147.x

[16] Svensson AM , Curry CJ , South ST , et al. Detection of a de novo interstitial 2q microdeletion by CGH microarray analysis in a patient with limb malformations, microcephaly and mental retardation. Am J Med Genet A. 2007;143A(12):1348‐1353. 17506097 10.1002/ajmg.a.31775

[17] Pescucci C , Caselli R , Grosso S , et al. 2q24‐q31 deletion: report of a case and review of the literature. Eur J Med Genet. 2007;50(1):21‐32. 17088112 10.1016/j.ejmg.2006.09.001

[18] József Z , Denis D . Synpolydactyly in mice with a targeted deficiency in the HoxD complex. Nature. 1996;384(6604):69‐71. 8900279 10.1038/384069a0

[19] Goodman FR , Majewski F , Collins AL , Scambler PJ . A 117‐kb microdeletion removing HOXD9– HOXD13 and EVX2 causes synpolydactyly. Am J Hum Genet. 2002;70(2):547‐555. 11778160 10.1086/338921 PMC384929

[20] Beccari L , Jaquier G , Lopez Delisle L , et al. Dbx2 regulation in limbs suggests interTAD sharing of enhancers. Dev Dynam. 2021;250(9):1280‐1299. 10.1002/dvdy.303 PMC8451760 33497014

[21] Sheth R , Marcon L , Bastida MF , et al. Hox genes regulate digit patterning by controlling the wavelength of a Turing‐type mechanism. Science. 2012;338(6113):1476‐1480. 23239739 10.1126/science.1226804 PMC4486416

[22] Del Campo M , Jones MC , Veraksa AN , et al. Monodactylous limbs and abnormal genitalia are associated with hemizygosity for the human 2q31 region that includes the HOXD cluster. Am J Hum Genet. 1999;65(1):104‐110. 10364522 10.1086/302467 PMC1378080

[23] Fantini S , Vaccari G , Brison N , Debeer P , Tylzanowski P , Zappavigna V . A G220V substitution within the N‐terminal transcription regulating domain of HOXD13 causes a variant synpolydactyly phenotype. Hum Mol Genet. 2008;18(5):847‐860. 19060004 10.1093/hmg/ddn410

[24] Kurban M , Wajid M , Petukhova L , Shimomura Y , Christiano AM . A nonsense mutation in the HOXD13 gene underlies synpolydactyly with incomplete penetrance. J Hum Genet. 2011;56(10):701‐706. 21814222 10.1038/jhg.2011.84 PMC4296310

[25] Zhang M , Lu L , Wei B , et al. Brachydactyly typeA3 is caused by a novel 13 bpHOXD13 frameshift deletion in a Chinese family. Am J Med Genet A. 2020;182(10):2432‐2436. 32789964 10.1002/ajmg.a.61788

[26] Spielmann M , Kakar N , Tayebi N , et al. Exome sequencing and CRISPR/Cas genome editing identify mutations ofZAK as a cause of limb defects in humans and mice. Genome Res. 2016;26(2):183‐191. 26755636 10.1101/gr.199430.115 PMC4728371

[27] Funk CR , Huey ES , May MM , et al. Rare missense variant p.Ala505Ser in the ZAK protein observed in a patient with split‐hand/foot malformation from a non‐consanguineous pedigree. J Int Med Res. 2020;48(4):1410458073. 10.1177/0300060519879293 PMC7144677 32266845

[28] Lézot F , Thomas BL , Blin‐Wakkach C , et al. Dlx homeobox gene family expression in osteoclasts. J Cell Physiol. 2010;223(3):779‐787. 20205208 10.1002/jcp.22095

[29] Theisen A , Rosenfeld JA , Shane K , et al. Refinement of the region for Split hand/foot malformation 5 on 2q31.1. Mol Syndromol. 2010;1(5):262‐271. 22140379 10.1159/000328405 PMC3214950

[30] Dai J , Kuang Y , Fang B , et al. The effect of overexpression of Dlx2 on the migration, proliferation and osteogenic differentiation of cranial neural crest stem cells. Biomaterials. 2013;34(8):1898‐1910. 23246068 10.1016/j.biomaterials.2012.11.051

[31] Elliott AM , Evans JA . The association of split hand foot malformation (SHFM) and congenital heart defects. Birth Defects Res A Clin Mol Teratol. 2008;82(6):425‐434. 18383509 10.1002/bdra.20452

[32] Kim K , Arima Y , Kitazawa T , et al. Endothelin regulates neural crest deployment and fate to form great vessels through Dlx5/Dlx6‐independent mechanisms. Mech Develop. 2013;130(11–12):553‐566. 10.1016/j.mod.2013.07.005 23933587

[33] Chauveau C , Bonnemann CG , Julien C , et al. Recessive TTN truncating mutations define novel forms of core myopathy with heart disease. Hum Mol Genet. 2014;23(4):980‐991. 24105469 10.1093/hmg/ddt494 PMC3954110

[34] Tharp CA , Haywood ME , Sbaizero O , Taylor MRG , Mestroni L . The giant protein Titin's role in cardiomyopathy: genetic, transcriptional, and post‐translational modifications of TTN and their contribution to cardiac disease. Front Physiol. 2019;10:1436. 31849696 10.3389/fphys.2019.01436 PMC6892752

[35] Monzen K , Hiroi Y , Kudoh S , et al. Smads, TAK1, and their common target ATF‐2 play a critical role in cardiomyocyte differentiation. J Cell Biol. 2001;153(4):687‐698. 11352931 10.1083/jcb.153.4.687 PMC2192375

[36] Karunakaran KB , Gabriel GC , Balakrishnan N , Lo CW , Ganapathiraju MK . Novel protein‐protein interactions highlighting the crosstalk between hypoplastic left heart syndrome, ciliopathies and neurodevelopmental delays. Genes (Basel). 2022;13(4):627. 35456433 10.3390/genes13040627 PMC9032108

[37] Traylor RN , Dobyns WB , Rosenfeld JA , et al. Investigation ofTBR1 hemizygosity: four individuals with 2q24 microdeletions. Mol Syndromol. 2012;3(3):102‐112. 23112752 10.1159/000342008 PMC3473348

[38] Miller AR , Hawkins NA , Mccollom CE , et al. Mapping genetic modifiers of survival in a mouse model of Dravet syndrome. Genes Brain Behav. 2014;13(2):163‐172. 24152123 10.1111/gbb.12099 PMC3930200

[39] Ben‐Shalom R , Keeshen CM , Berrios KN , An JY , Sanders SJ , Bender KJ . Opposing effects on Na V 1.2 function underlie differences between SCN2A variants observed in individuals with autism spectrum disorder or infantile seizures. Biol Psychiatry. 2017;82(3):224‐232. 28256214 10.1016/j.biopsych.2017.01.009 PMC5796785

